# Transient Receptor Potential Canonical Type 3 Channels Control the Vascular Contractility of Mouse Mesenteric Arteries

**DOI:** 10.1371/journal.pone.0110413

**Published:** 2014-10-13

**Authors:** Soo-In Yeon, Joo Young Kim, Dong-Soo Yeon, Joel Abramowitz, Lutz Birnbaumer, Shmuel Muallem, Young-Ho Lee

**Affiliations:** 1 Department of Physiology and BK21 PLUS Project for Medical Sciences, Yonsei University College of Medicine, Seoul, Korea; 2 Department of Pharmacology and BK21 PLUS Project for Medical Sciences, Yonsei University College of Medicine, Seoul, Korea; 3 Department of Physiology, Kwandong University College of Medicine, Gangneung, Korea; 4 Laboratory of Neurobiology, National Institute of Environmental Health Sciences, Research Triangle Park, North Carolina, United States of America; 5 Epithelial Signaling and Transport Section, Molecular Physiology and Therapeutics Branch, National Institute of Dental and Craniofacial Research, National Institutes of Health, Bethesda, Maryland, United States of America; SUNY College of Nanoscale Science and Engineering, United States of America

## Abstract

Transient receptor potential canonical type 3 (TRPC3) channels are non-selective cation channels and regulate intracellular Ca^2+^ concentration. We examined the role of TRPC3 channels in agonist-, membrane depolarization (high K^+^)-, and mechanical (pressure)-induced vasoconstriction and vasorelaxation in mouse mesenteric arteries. Vasoconstriction and vasorelaxation of endothelial cells intact mesenteric arteries were measured in TRPC3 wild-type (WT) and knockout (KO) mice. Calcium concentration ([Ca^2+^]) was measured in isolated arteries from TRPC3 WT and KO mice as well as in the mouse endothelial cell line bEnd.3. Nitric oxide (NO) production and nitrate/nitrite concentrations were also measured in TRPC3 WT and KO mice. Phenylephrine-induced vasoconstriction was reduced in TRPC3 KO mice when compared to that of WT mice, but neither high K^+^- nor pressure-induced vasoconstriction was altered in TRPC3 KO mice. Acetylcholine-induced vasorelaxation was inhibited in TRPC3 KO mice and by the selective TRPC3 blocker pyrazole-3. Acetylcholine blocked the phenylephrine-induced increase in Ca^2+^ ratio and then relaxation in TRPC3 WT mice but had little effect on those outcomes in KO mice. Acetylcholine evoked a Ca^2+^ increase in endothelial cells, which was inhibited by pyrazole-3. Acetylcholine induced increased NO release in TRPC3 WT mice, but not in KO mice. Acetylcholine also increased the nitrate/nitrite concentration in TRPC3 WT mice, but not in KO mice. The present study directly demonstrated that the TRPC3 channel is involved in agonist-induced vasoconstriction and plays important role in NO-mediated vasorelaxation of intact mesenteric arteries.

## Introduction

Changes in intracellular calcium concentration ([Ca^2+^]_i_) lead to diverse cellular responses, including vasoconstriction and release of vasodilators such as nitric oxide (NO) from endothelial cells (ECs). Vascular contractility is primarily mediated by [Ca^2+^]_i_ increase in response to agonists or mechanical stimulation [1]. The elevation in [Ca^2+^]_i_ results in activation of the Ca^2+^/calmodulin-dependent enzyme myosin light chain kinase and the subsequent phosphorylation of regulatory myosin light chains (MLC_20_). This increased MLC_20_ phosphorylation enhances actomyosin ATPase activity and induces vascular contraction [2]. ECs also produce and release vasoactive substances, such as nitric oxide (NO), to regulate vascular contractility. It is well documented that Ca^2+^-dependent mechanisms initiate the production and release of NO. The elevation of [Ca^2+^]_i_ in ECs activates nitric oxide synthase (NOS) via the Ca^2+^/calmodulin complex, which catalyzes the production of NO [3], [4]. In non-excitable ECs, the increase in [Ca^2+^]_i_ mainly depends on Ca^2+^ influx through store-operated Ca^2+^ channels and non-selective Ca^2+^ channels [5].

Transient receptor potential (TRP) channels are non-selective cation channels [6] that directly act as Ca^2+^ entry channels in the plasma membrane or change membrane potentials, thereby modulating the driving forces for Ca^2+^ entry, to regulate [Ca^2+^]_i_ [7], [8]. In mammals, almost 30 members of the TRP family have been discovered [9], [10]. Among the subfamilies of TRP channels, canonical TRPs (TRPCs), including TRPC3, are expressed in smooth muscle and endothelial cells [11]–[16]. TRPC3 functions as both a receptor-operated Ca^2+^entry channel and a store-operated channel [6], [13], [17]–[19]. Therefore, TRPC3 channels may play an important role in agonist-induced contraction and production of NO.

Several studies have described the role of TRPC3 channels in vascular contractility. It was shown that antisense suppression of TRPC3 significantly attenuated UTP-induced membrane depolarization and constriction [13]. It was also reported that TRPC3 channels are upregulated in TRPC6-deficient smooth muscle cells, and increased TRPC3 enhanced systemic blood pressure and agonist-induced contraction in intact cerebral arteries [20]. Additionally, high levels of TRPC3 protein are expressed in spontaneously hypertensive rats (SHR), as well as in patients with hypertension, and upregulated TRPC3 increased the Ca^2+^ influx in SHRs compared with normotensive Wistar-Kyoto rats (WKY) [21]–[24]. The accumulated evidence suggests that TRPC3 channels may play an important role in agonist-induced contraction and cardiovascular disease, especially hypertension. On the other hand, TRPC3 channels are recognized as important Ca^2+^-permeable cation channels in NO production. TRPC3 involvement has been shown in flow- and bradykinin-induced vasodilation in rat small mesenteric arteries, probably by mediating the Ca^2+^ influx into ECs [12], [25]. TRPC3 was also shown to participate in EC Ca^2+^ influx and vasorelaxation of the aorta [26].

Despite several years of study regarding the possible role of TRPC channels as store-operated Ca^2+^ channels and receptor-operated Ca^2+^ channels, surprisingly little information exists regarding their role in the mechanisms of vasoconstriction and vasorelaxation in intact arteries. Because selective pharmacological inhibitors for TRPC3 are not available, the use of gene knockout (KO) mice offers a good option for studying specific roles of TRPC in vascular contractility regulation. In the present study, we investigated the role of the TRPC3 channel in the agonist (phenylephrine)-, mechanical (pressure)-, and membrane depolarization (high K^+^)-induced vasoconstriction of mouse ECs intact mesenteric arteries from TRPC3 KO mice. We also determined the functional role of TRPC3 in endothelium-dependent vasorelaxation. We show here that the TRPC3 channel is involved in agonist-induced vasoconstriction and critically functions in NO-mediated vasorelaxation of ECs intact mesenteric arteries.

## Materials and Methods

This investigation conforms to the *Guide for the Care and Use of Laboratory Animals*, published by the US National Institutes of Health (NIH publication No. 85–23, revised 1996). The experimental protocols used in this study were reviewed and approved by the Ethic Committee, Institutional Animal Care and Use Committee of Yonsei University Health System.

### Animal model

Generation of TRPC3 KO mice has been described previously [27]. TRPC KO mice were provided by Dr. JY Kim. TRPC3 littermates were analyzed by PCR, using the genomic DNA extracted from the tails of mice as templates and the forward and reverse primer pair. Primer sequences were as follows: mrTRPC3.s, 5′-ATT CTT CGA AGC CCC TTC AT-3′; mrTRPC3.as, 5′-CTC CTT GCA CTC AGA CCA CA-3′.

### Tissues preparation and EC bENd.3 cells

Six to eight week-old TRPC3 KO and age-matched WT mice, as controls, were euthanized using CO_2_ gas. The depth of anaesthesia was evaluated by pinching the animal’s paw with forceps. The mesenteric arteries were removed and immersed in normal Krebs-Henseleit (KH) solution composed of (in mmol/L): NaCl (119), CaCl_2_(2.5), NaHCO_3_(25), MgSO_4_(1.2), KH_2_O_4_(1.2), KCl (4.6), and glucose (11.1). The KH solution was continuously aerated with a 95% O_2_ and 5% CO_2_ gas mixture. Second-order mesenteric arteries (2∼3 mm long) were carefully dissected from surrounding adipose tissue under a dissecting microscope (Nikon, Tokyo, Japan).

Mouse endothelial cell bEnd.3 cells were used in endothelial [Ca^2+^]_i_ measurement experiments. The bEnd.3 cell line (American Type Culture Collection, Manassas, VA, USA) is an immortalized mouse cell line from brain capillary endothelial cells. Cells were grown in Dulbecco’s modified Eagle’s medium (DMEM; Gibco Invitrogen, Carlsbad, CA, USA) containing high glucose concentration (4.5 g/L) and 10% fetal bovine serum (FBS; Gibco Invitrogen). Cells were cultured in a humidified cell culture incubator at 37°C and an atmosphere of 5% CO_2_/95% air. The bEnd.3 cells used in this study were passaged between 12 to 20 times.

### Arteriograph-derived diameter measurement, as an indicator of vasoconstriction, vasorelaxation, and pressure-induced myogenic tone

The diameter measurement methods using an arteriograph system were described in a previous study [28], [29]. Mesenteric artery segments (100–180 µm inner diameter and 2–3 mm in length) were dissected and cannulated in a pressure myograph (Living Systems Instrumentation, Burlington, VT, USA), filled with KH solution, and subsequently placed on the stage of an inverted microscope (Eclipse TS100/TS100-F, Nikon Inc., Melville, NY, USA). The proximal cannula was connected to a solid-state pressure transducer and a reservoir of KH solution, and the intraluminal pressure was controlled by a pressure servo-controller to adjust the transmural pressures. The distal cannula was connected to the luer-lock valve, which was opened to flush the lumen during the initial cannulation. After cannulation, the valve was closed, and all measurements were conducted under no-flow conditions. The arterial lumen diameter was recorded using the Soft Edge Acquisition Subsystem (IonOptix, Milton, MA, USA).

The KH perfusion and superfusion of the arterial segments was equilibrated with a 95% O_2_ and 5% CO_2_ gas mixture at 37°C. After mounting, the mesenteric arterial segments were stretched longitudinally to the approximate *in situ* length and were maintained at a 40 mmHg transmural pressure for a 40–60 min equilibration period.

In the present study, ECs intact arteries were used in all experiments. Endothelial function was checked at the beginning of each experiment with 1 µM acetylcholine in arteries that were precontracted using 70 mM K^+^ solution (equimolar substitution of Na^+^ with K^+^).

After equilibration, to compare vasoconstriction between TRPC KO and WT mice, the cumulative concentration-response curves of phenylephrine (10 nmol/L to 10 µmol/L) and 70 mM K^+^ solution-induced vasoconstriction were measured in vessels of TRPC3 KO and age-matched WT mice.

To compare vasorelaxation between TRPC KO and WT mice, the cumulative concentration-response curves of acetylcholine (10 nmol/L to 100 µmol/L) were evaluated in arteries precontracted with phenylephrine (10 µmol/L). For sodium nitroprusside, we examined the effect of a single concentration (10 µmol/L). We also examined the effect of pyrazole-3 (ethyl-1-(4-(2,3,3-trichloroacrylamide)phenyl)-5-(trifluoromethyl)-1H-pyrazole-4-carboxylte, 3 µmol/L), a selective TRPC3 blocker, on acetylcholine-induced vasorelaxation in TRPC WT mice.

In vasoconstriction and vasorelaxation experiments, artery diameter changes are expressed as a percentage of the maximal dilation achieved by replacing the control KH solution with Ca^2+^-free KH solution at the end of the experiment.

Pressure-induced myogenic tone was measured in mesenteric arteries of TRPC KO and WT mice as previously described [28]. After being mounted and equilibrated at a 40 mmHg intraluminal pressure, the pressure was increased in a stepwise manner from 20 to 140 mmHg in 20 mmHg increments, and each pressure was maintained for 10 min to allow the blood vessel diameter to stabilize before measurement. After a series of step changes, the transmural pressure was returned to 40 mmHg, and the vessel was allowed to re-equilibrate for minimum of 40 min. At the end of each experiment, a passive pressure-diameter relationship was established in Ca^2+^-free KH solution containing 0.1 µmol/L nifedipine to determine the maximum passive diameter. Myogenic tone was calculated s a percent decrease in diameter from fully relaxed diameter in Ca^2+^-free KH solution with nifedipine by the equation: [1−(D_A_/D_P_)]×100%, where D_A_ is the active diameter at a given pressure in normal KH solution in the presence of extracellular Ca^2+^ and D_P_ is the passive diameter at a given pressure in Ca2+-free KH solution with 0.1 µmol/L nifedipine.

### Simultaneous measurement of [Ca^2+^]_i_ and diameter in pressurized arteries

Changes in [Ca^2+^]_i_ and diameter in the pressurized arteries of TRPC KO and WT mice were measured as previously described [28]. Mesenteric artery segments were loaded with the Ca^2+^-sensitive fluorescent indicator Fura-2AM (10 µmol/L; Molecular Probes, Eugene, OR, USA) and 0.02% Cremophor EL (Sigma, St Lois, MO, USA) in KH as previously reported [30]. Arteries were incubated in this solution for 3 h at room temperature in the dark. Fura-2AM-loaded mesenteric artery segments were mounted in a pressure myograph, pressurized to 40 mmHg using a pressure servo-controller, and then superfused with KH (37°C) that was aerated with 95% O_2_ and 5% CO_2_ to wash out excess dye and allow for hydrolysis of acetoxymethyl ester (AM) groups by intracellular esterases. Fura-2AM-loaded vessels were alternately excited at 340 and 380 nm at a frequency of 1 Hz with an IonOptix Hyperswitch dual excitation light source, and the respective 510 nm emissions were detected with a photomultiplier tube. Background-subtracted 340/380 emission ratios (R340/380) were calculated with IonOptix Ion Wizard software and recorded continuously throughout the experiment. The fluorescence emission at 510 nm (R340/380) and the changes in arterial diameter, monitored by videomicroscopy (IonOptix), were recorded simultaneously.

In the present study, changes in smooth muscle [Ca^2+^]_i_ and diameter upon application of acetylcholine (10 µmol/L) was recorded simultaneously in precontracted arteries of TRPC KO and WT mice treated with phenylephrine (10 µmol/L).

### Measurement of [Ca^2+^]_i_ ratio in bEnd.3 cells

The [Ca^2+^]_i_ increase in response to acetylcholine treatment (10 µmol/L) was measured in mouse endothelial bEnd.3 cells in the absence and presence of pyrazole-3 (3 µmol/L) using ratiometric imaging system (Nippon Roper, Tokyo, Japan).

Cells were placed on coverslips in 24-well plates and loaded with 5 µmol/L Fura-2AM for 20 min at 37°C in serum free medium. After incubation, the coverslips were placed into dishes and perfused with PSS. The endothelial Ca^2+^ ratio was determined by measuring the fluorescence of Fura-2AM at excitation of 340 and 380 nm, and the emission at 510 nm was simultaneously monitored using the Ratiometric Imaging System (Model Cascade 512B Set, Nippon Roper).

### Detection of NO fluorescence using 4,5-diaminofluorescence-2 diacetate

NO production in mesenteric arteries was measured using DAF-2 DA (Santa Cruz Biotech, Dallas, TX, USA), a specific NO indicator. After equilibration for 30–40 min, DAF-2DA (1 µmol/L) and acetylcholine (10^−5^ mol/L) were added to each artery for 30 min at 37°C in KH solution, and the arteries were then washed with KH solution. Fluorescent images at 480 nm excitation were detected on a fluorescent inverted microscope (IX71/DP71, Tokyo, Japan). All images were acquired and analyzed using the Metamorph software (Molecular Devices, Sunnyvale, CA, USA).

### Determination of nitrate/nitrite concentration using a colorimetric assay kit

The concentrations of nitrate/nitrite (NO metabolites) were determined using a commercially available colorimetric assay (Sigma) according to the manufacturer’s instructions. Briefly, isolated mesenteric arteries were stimulated with acetylcholine (10 µmol/L) for 24 h. To measure nitrate/nitrite concentrations, conditioned medium was collected and incubated at room temperature with nitrate reductase and enzyme cofactor, and Griess reagent A and B were added in consecutive order. The absorbance of the mixture at 540 nm was determined with a microplate reader (VERSAmax, Molecular devices, Union City, USA).

### Hematoxylin and Eosin staining

Mesenteric arteries of TRPC3 WT and KO mice were carefully isolated and then fixed with 4% paraformaldehyde for 1 h. Samples were embedded in OCT compound and 10-µm-thick sections were cut for hematoxylin and eosin staining. The slides were examined using a light microscope (BX51/BX61, Olympus, Tokyo, Japan).

### Statistics

All values given in the text are expressed as mean±SEM and were analyzed by two-way ANOVA, followed by the Student-Newman-Keuls post hoc test. Differences were considered significant if *P*<0.05.

## Results

### Role of TRPC3 in vasoconstriction

**Figure 1:**
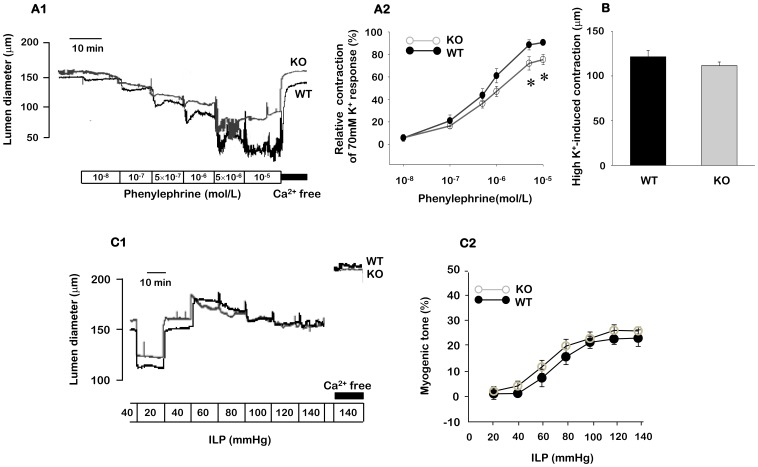
The effect of TRPC3 KO on vasoconstriction of ECs intact mesenteric arteries. A1. Representative recordings of the differences in phenylephrine-induced vasoconstriction between WT and TRPC3 KO mice. A2. Mean data for phenylephrine-induced vasoconstriction in WT and TRPC3 KO mice (n = 8–9). B. Mean data for 70 mmol/L KCl-induced vasoconstriction in WT and TRPC3 KO mice (n = 12–14). Vessel diameter was measured at an intraluminal pressure of 40 mmHg, C1. Representative recordings of the differencesin pressure-induced myogenic tone between WT and TRPC3 KO mice. C2. Mean data for pressure-induced myogenic tone in WT and TRPC3 KO mice (n = 6–8). Data are expressed as mean ± SEM. *P<0.05. ILP: intraluminal pressure.

To study if TRPC3 channels affect vasoconstriction, we compared vascular responsiveness to agonist (phenylephrine)-, membrane depolarization (high K^+^)-, and mechanical (pressure)-induced vasoconstriction between ECs intact mesenteric arteries of TRPC3 WT and KO mice. We first examined the effect of TRPC3 on phenylephrine-induced vasoconstriction in mesenteric arteries from TRPC3 WT and KO mice. As shown in Fig. 1A, phenylephrine induced concentration-dependent vasoconstriction. Phenylephrine-induced vasoconstriction was decreased in TRPC3 KO mice compared to TRPC3 WT mice. The magnitude of the 10 µmol/L phenylephrine-induced vasoconstriction in TRPC3 WT and KO mice was 90.6±2.3% and 75.4±4.6% (n = 8–9), respectively. In response to 70 mmol/L KCl-induced vasoconstriction, there was no difference between TRPC3 WT and KO mice (Fig. 1B, n = 12–14).

To evaluate the role of TRPC3 in myogenic tone, we evaluated pressure-induced myogenic tone in ECs intact mesenteric arteries of TRPC3 WT and KO mice. When intraluminal pressure was increased in a stepwise manner from 20 to 140 mmHg in 20-mmHg increments, mesenteric arteries constricted in response to an increase in intraluminal pressure similarly between TRPC3 WT and KO mice (Fig. 1C, n = 6–8).

### Role of TRPC3 in vasorelaxation

**Figure 2:**
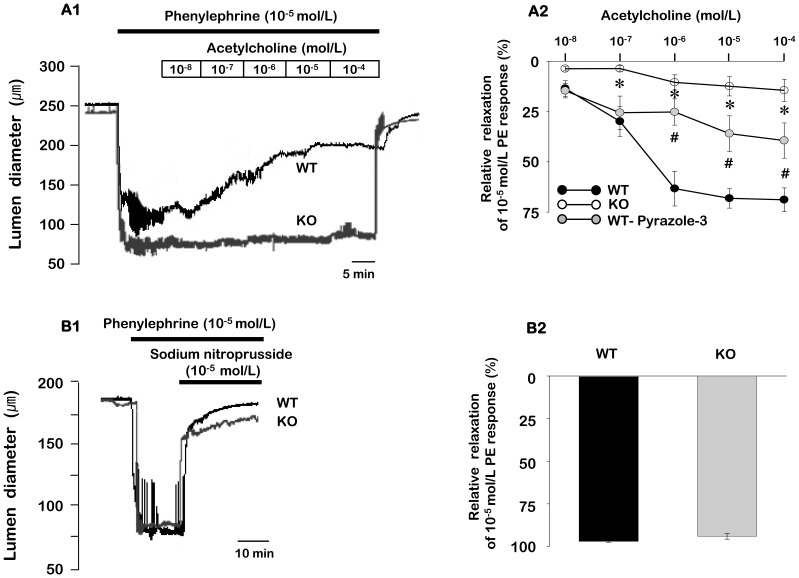
The effect of TRPC3 KO on vasorelaxation of ECs intact mesenteric arteries. A1. Representative recordings of vasorelaxation in response to acetylcholine in WT and KO mice. A2. Mean data for vasorelaxation in response to acetylcholine in WT and TRPC3 KO mice, and the effect of pyrazole- 3 on acetylcholine-induced vasorelaxation in WT mice. Pyrazole-3 was administered 30 min before application of phenylephrine (PE). B1. Representative recordings of vasorelaxation in response to sodium nitroprusside in WT and KO mice. B2. Mean data for vasorelaxation in response to sodium nitroprusside in WT and TRPC3 KO mice. Data are expressed as mean ± SEM (n = 4–6) and are normalized to the maximal vasoconstriction to phenylephrine (10 µmol/L). *P<0.005 and #*P*<0.05, compared to WT mice.

We compared endothelium dependent- and independent-vasorelaxation between TRPC3 WT and KO mice. To determine the role of TRPC3 in endothelium-dependent relaxation, we compared acetylcholine-induced relaxation in phenylephrine precontracted ECs intact arteries from TRPC3 WT and KO mice (Fig. 2A). Acetylcholine produced concentration-dependent vasorelaxation that was significantly reduced in TRPC3 KO mice compared to WT mice (n = 11). We additionally examined the contribution of TRPC3 to the endothelium-dependent relaxation using the selective TRPC3 blocker pyrazole-3. Treatment with 3 µmol/L pyrazole-3 resulted in significant inhibition of the acetylcholine-induced vasorelaxation in the mesenteric arteries of TRPC3 WT mice (n = 7).

To examine endothelium-independent relaxation, we also compared sodium nitroprusside-induced relaxation in phenylephrine precontracted ECs intact arteries from TRPC3 WT and KO mice (Fig. 2B). However, there was no significant difference between TRPC3 WT and KO mice. The mean vasorelaxation induced by 10 µmol/L sodium nitroprusside was 97.0±0.5% and 94.0±1.7% (n = 4–6) for TRPC3 WT and KO mice, respectively.

**Figure 3:**
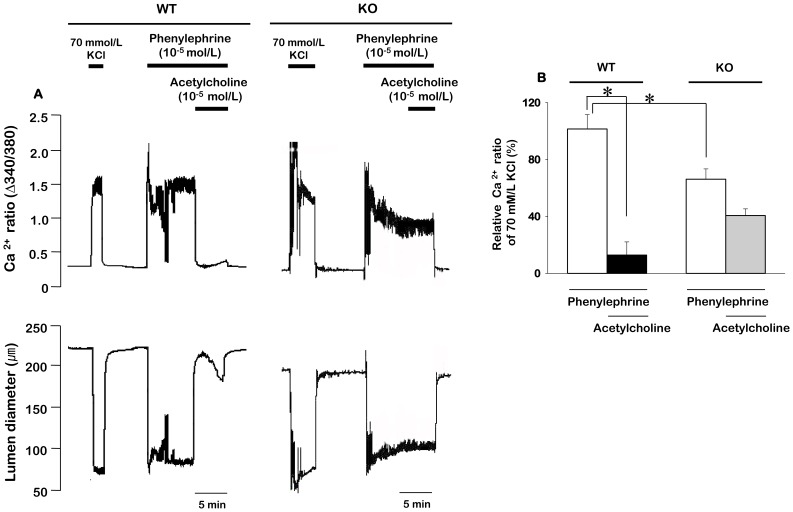
Changes in the [Ca^2+^]_i_ and the vessel diameter induced by acetylcholine treatment in phenylephrine precontracted ECs intact mesenteric arteries from WT and TRPC3 KO mice. A. Representative recordings of changes in the [Ca^2+^]_i_, expressed as the ratio of 510 nm emission fluorescence evoked by 340 and 380 nm excitation light (380/340 [Ca^2+^]_i_ ratio) and the diameter in response to acetylcholine in phenylephrine precontracted arteries. B. Statistical analysis of changes in the [Ca^2+^]_i_ ratio induced by acetylcholine treatment between WT and TRPC3 KO mice. Data are expressed as mean ± SEM (n = 5–6) and relative percentage of the 70 mmol/L KCl-induced [Ca^2+^]_i_ ratio. *P<0.05.

Because Ca^2+^ plays a critical role in the regulation of vascular tone, we compared the effect of acetylcholine on phenylephrine-induced changes in Ca^2+^ ratio and vessel diameter between ECs intact mesenteric arteries of TRPC3 WT and KO mice. As shown in Fig. 3, the addition of 10 µmol/L acetylcholine inhibited the phenylephrine-induced increase in Ca^2+^ ratio and subsequent relaxation of phenylephrine-constricted arteries in TRPC3 WT mice. However, acetylcholine had little effect on the increase in Ca^2+^ ratio and vasoconstriction evoked by stimulation with 10 µmol/L phenylephrine in TRPC3 KO mice. We also compared the 10 µmol/L phenylephrine-induced Ca^2+^ ratio between WT and TRPC3 KO mice. Phenylephrine-induced Ca^2+^ ratio was significantly decreased in TRPC3 KO mice compared to TRPC3 WT mice. In the TRPC3 WT mice, the amplitude of the phenylephrine-induced Ca^2+^ ratio was 93.4±8.2% (n = 6) in the absence of acetylcholine and 12.6±6.2% (n = 6) in the presence of acetylcholine. However, in the TRPC3 KO mice, the amplitude of the phenylephrine-induced Ca^2+^ ratio was 64.2±6.0% (n = 5) in the absence of acetylcholine and 43.9±4.9% (n = 5) in the presence of acetylcholine.

### Role of TRPC3 in the [Ca^2+^]_i_ response to acetylcholine in bEnd.3 mouse endothelial cells

**Figure 4:**
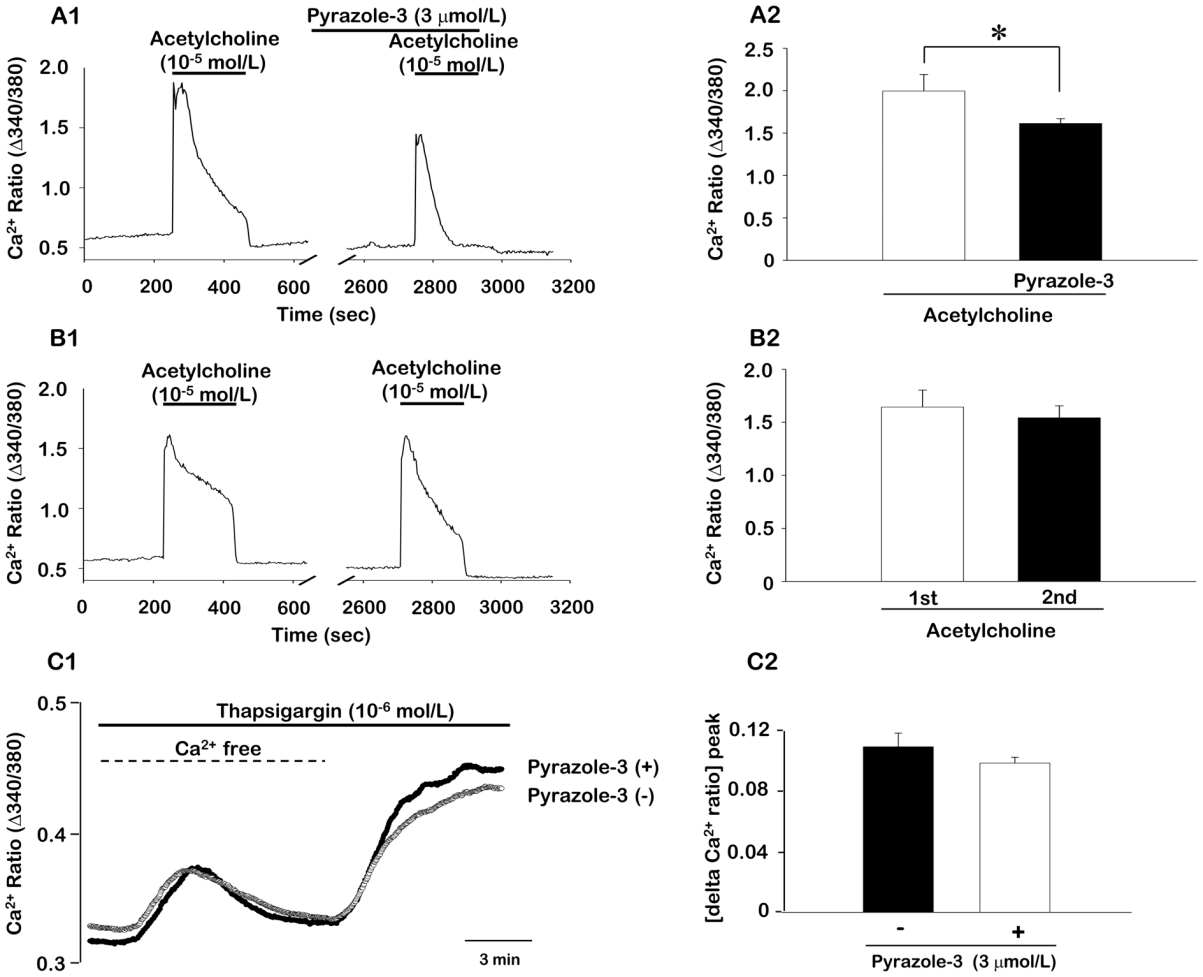
The effect of the TRPC3 channel blocker pyrazole-3 on the acetylcholine-induced [Ca^2+^]_i_ increase and store-operated Ca^2+^ entry in bEnd3 mouse endothelial cells. A1 and A2. Representative recordings (A1) and mean data (A2) of the effect of pyrazole-3 on the acetylcholine-induced increase in [Ca^2+^]_i_. 3 µmol/L pyrazole-3 was added 30 min before the application of 10 µmol/L acetylcholine. B1 and B2. Differences in [Ca^2+^]_i_ between two consecutive challenges of acetylcholine. Data are expressed as mean ± SEM (n = 17–23). *P<0.05. C1 and C2. Store operated Ca^2+^ entry in the absence and presence of pyrazole-3 of bEnd3 mouse endothelial cells. C1. Representative recordings of Ca^2+^ ratio in fluorescence spectrometry before, during and after Ca^2+^ depletion with subsequent addition of thapsigargin (1 µmol/L) in the absence and presence of purazole-3. C2. mean data (n = 19) of peak increase of Ca^2+^ ratio following readdition of extracellular Ca^2+^ in the absence and presence of pyrazole-3.

We evaluated the specific role of endothelial cell TRPC3 in the acetylcholine-induced increase in [Ca^2+^]_i_ using bEnd.3 mouse endothelial cells. As shown in Fig. 4, the application of 10 µmol/L acetylcholine to endothelial cells caused a significant, rapid rise in [Ca^2+^]_i_, followed by a secondary sustained [Ca^2+^]_i_ plateau (Fig. 4A). Exposure of endothelial cells in the resting state to 3 µmol/L pyrazole-3 did not change the resting level of [Ca^2+^]_i_. However, the acetylcholine-induced rapid increase in [Ca^2+^]_i_ and secondary sustained plateau were significantly inhibited by pre-incubation With pyrazole-3. In a control experiment, we measured differences in [Ca^2+^]_i_ with two consecutive challenges of acetylcholine. There was little difference in the magnitude o the Ca^2+^ response with two consecutive challenges with acetylcholine (Fig. 4B).

We also determined the function of TRPC3 as a store-operated Ca^2+^ entry (SOCE) channel using store-depleting sarco−/endoplasmic reticulum Ca^2+^-ATPase (SERCA) inhibitor thapsigargin. The Fura-2-fluorescence ratio prior to extracellular Ca^2+^ removal was similar in the absence and presence of 3 µmol/L pyrazole-3 of bEnd.3 mouse endothelial cells (Fig. 4C). Addition of the thapsigargin (1 µmol/L) triggered release of Ca^2+^ from intracellular stores, leading to rapid, transient increase in cytosolic Ca^2+^. As illustrated in Fig. 4C, the subsequent addition of extracellular Ca^2+^ was followed by a rapid increase of Fura-2-fluorescence in both cell types reflecting SOCE. In the presence of pyrazole-3, the peak of SOCE was partially reduced, but not significantly and the slope of SOCE was similar in the absence and presence of pyrazole-3.

### Role of TRPC3 in NO production

**Figure 5:**
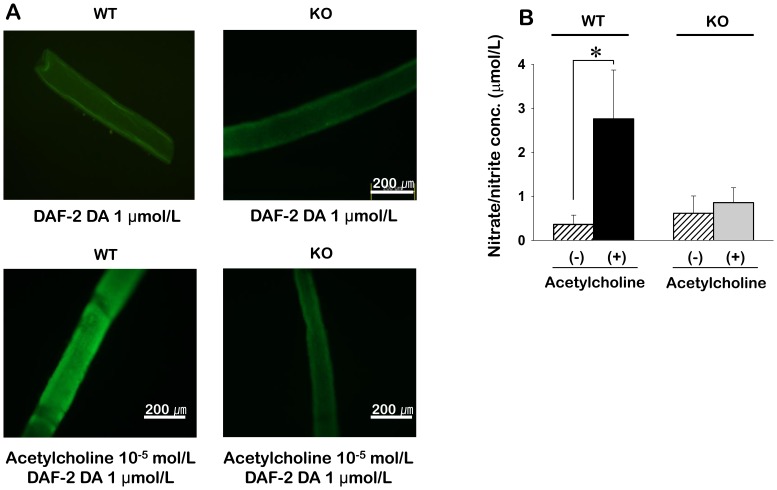
NO production by ECs intact mesenteric artery fragments from WT and TRPC3 KO mice. A. The effect of acetylcholine on DAF-2 DA fluorescence intensity in mesenteric arteries from WT and KO mice. B. The effect of acetylcholine on nitrate/nitrite concentration in arteries from WT and KO mice (n = 4). *P<0.05.

To determine if the decrease in vasorelaxation in TRPC3 KO mice was caused by decreased NO production, we measured NO production using DAF-2DA, a nitric oxide-specific fluorescent indicator (Fig. 5). Acetylcholine elicited NO production from the endothelium of TRPC3 WT controls, with DAF-2 DA fluorescence intensity increasing by 18-fold in the arteries treated with acetylcholine compared to untreated arteries. However, in TRPC3 KO mice, acetylcholine had little effect on DAF-2 DA fluorescence intensity. We also measured nitrate/nitrite concentration as an indicator of NO production. When acetylcholine was added to arteries from TRPC3 WT mice, nitrate/nitrite concentration was increased to 2.8±1.1 µmol/L (n = 4) from the untreated level of 0.4±0.2 µmol/L (n = 4). However, there was no change in nitrate/nitrite concentration in response to acetylcholine in arteries for TRPC3 KO mice.

## Discussion

In the present study, we determined the role of TRPC3 channels in vascular contractility using TRPC3 KO mice and demonstrated two major findings. First, TRPC3 channels may play an important role in agonist-induced contraction. We demonstrated that phenylephrine-induced vasoconstriction is decreased in TRPC3 KO mice when compared to that of WT mice, but neither membrane depolarization (high K^+^)- nor mechanical (pressure)-induced vasoconstriction were altered in TRPC3 KO mice. Secondly, endothelial TRPC3 contributes to endothelial NO release and subsequent endothelium-dependent vasorelaxation, which was evidenced as follows: (1) acetylcholine-induced vasorelaxation was inhibited in TRPC3 KO mice and by the selective TRPC3 blocker, pyrazole-3; (2) acetylcholine inhibited the phenylephrine-induced increase in [Ca^2+^]_i_ ratio and subsequent relaxation in vessels from TRPC3 WT mice, but had little effect on those parameters in KO mice; (3) acetylcholine induced increases in [Ca^2+^]_i_ in endothelial cells, which was inhibited by pyrazaole-3; (4) NO was released from mesenteric artery segments from WT mice in response to acetylcholine, but not from vessels from KO mice.

### Role of TRPC3 in vasoconstriction

TRPC channels are Ca^2+^-permeable non-selective cation channels in vascular cells [6]. Physiologically, Ca^2+^ influx through TRPCs leads to an [Ca^2+^]_i_ elevation that drives vascular smooth muscle cell contraction, leading to vasoconstriction of resistance arteries and thus, elevated peripheral resistance and blood pressure [20], [31]. In the present study, we investigated the role of TRPC3 in vasoconstriction of resistance arteries, such as the mesenteric arteries, under in response to agonist (phenylephrine), membrane depolarization (high K^+^), and mechanical (pressure) changes. In phenylephrine-induced vasoconstriction, we demonstrated phenylephrine-induced vasoconstriction is inhibited in TRPC3 KO mice when compared to that of WT mice. We also demonstrated phenylephrine-induced Ca^2+^ ratio was significantly decreased in TRPC3 KO mice compared to TRPC3 WT mice. These results suggest that TRPC3 plays an important role in receptor-mediated contractile mechanisms [13], [32], [33]. However, membrane depolarization-induced vasoconstriction, using high K^+^ concentrations, was not inhibited in TRPC3 KO mice. We also investigated the role of TRPC3 in pressure-induced myogenic tone and demonstrated that there was no significant difference between TRPC3 WT and KO mice. Our results suggest that TRPC3 is not important for membrane depolarization-induced contraction and involved in pressure-induced myogenic tone. Recently identified mammalian TRP channels are candidates for mechanosensory functions in arterial smooth muscle [34], [35]. Two members of the TRP family, TRPC6 and TRPM4, are considered mediators of pressure-induced myogenic constriction in cerebral vessels [36], [37]. Although there are many previous reports about the role of TRPC3 in agonist-induced vasoconstriction, no previous reports have elucidated the role of TRPC3 channels in other methods of vasoconstriction. Therefore, our results provide more extensive experimental evidence for the role of TRPC3 channels in vasoconstriction.

### Role of TRPC3 in endothelium-dependent vasorelaxation

Because TRPC channels may be store-operated Ca^2+^ channels as well as receptor-operated Ca^2+^ channels, these channels are believed to promote vasorelaxation through increased endothelial Ca^2+^ and subsequent activation of NO. In fact, it was reported that TRPC channels are expressed in endothelial cells and contribute to endothelial cell Ca^2+^ regulation [38], [39]. It was also reported that TRPC3 participate in endothelial cell Ca^2+^ influx and vasorelaxation of the aorta [26]. This evidence suggests that TRPC3 channels may function in endothelium-dependent vasorelaxation. It is well known that conduit arteries, such as the aorta, contribute to an increase in systolic blood pressure and pulse wave velocity [40], and increased vasoconstriction of conduit arteries contributes to the production and/or maintenance of hypertension. However, resistance arteries, such as the mesenteric arteries, also play important roles in hypertension, especially in relation to increased peripheral resistance. Therefore, the role of TRPC3 in vasoconstriction and vasorelaxation in resistance arteries needs to be determined. In experiments performed with resistance arteries, TRPC3 was shown to be involved in flow- and bradykinin-induced vasodilation in rat small mesenteric arteries, probably by mediating the Ca^2+^ influx into endothelial cells [12], [25]. However, the previous findings measured only the effect of TRPC3 antisense oligonucleotides on the bradykinin-induced vasorelaxation and endothelial [Ca^2+^]_i_. Senadheera et al (2012) reported that the importance of TRPC3 in endothelium-dependent vasorelaxation due to endothelium-derived hyperpolarization (EDH) in mesenteric artery using TRPC3 KO mice [41]. Senadheera et al (2012) pointed the Ca^2+^ influx via TRPC3 of endothelial cells plays important role in EDH through K_Ca_ (Ca^2+^-activated K^+^ channel) activation. Although Senadheera et al (2012) pointed the role of Ca^2+^ influx via TRPC3 in endothelium-dependent vasorelaxation, further studies were required to determine the role of TRPC3 in NO production and NO-dependent vasorelaxation. In order to provide conclusive experimental evidence that TRPC3 channels function in endothelium-dependent vasorelaxation, in the present study, we examined the following: (1) the comparison of acetylcholine-induced vasorelaxation between TRPC3 WT and KO mice, (2) the effect of the selective TRPC3 blocker pyrazole-3 on acetylcholine-induced vasorelaxation in TRPC3 WT mice, (3) the comparison of the acetylcholine effect on the phenylephrine-induced increase in [Ca^2+^]_i_ ratio and vasoconstriction between TRPC3 WT and KO mice, (4) the changes in endothelial [Ca^2+^]_i_ in response to treatment with acetylcholine and pyrazole-3 and (5) the comparison of acetylcholine-induced NO release and nitrate/nitrite concentration between TRPC3 WT and KO mice.

In the present study, we demonstrated that acetylcholine-induced vasorelaxation is inhibited in TRPC3 KO mice and by pyrazole-3. We also demonstrated that acetylcholine inhibited the phenylephrine-induced increase in [Ca^2+^]_i_ ratio and subsequent relaxation in TRPC3 WT mice, but had little effect on phenylephrine-induced increase in [Ca^2+^]_i_ ratio and vasoconstriction in KO mice. These results suggest that TRPC3 contributes to the endothelium-dependent vasorelaxation, consistent with previous findings [12], [25]. It was well known that endothelium-dependent vasorelaxation is mediated by an increase in endothelial Ca^2+^ concentration, with subsequent production of NO [3], [4]. To further confirm the role of TRPC3 in endothelium-dependent vasorelaxation, we measured the changes in endothelial [Ca^2+^]_i_ and NO concentration in response to acetylcholine. We demonstrated that acetylcholine treatment increased [Ca^2+^]_i_ in bEnd.3 mouse endothelial cells, and this increase in [Ca^2+^]_i_ was inhibited by pyrazole-3. We also demonstrated that NO production in response to acetylcholine was increased in TRPC3 WT mice, but not in KO mice. Acetylcholine also increased the nitrate/nitrite production by mesenteric arteries of WT mice, but not of TRPC3 KO mice.

In the present study, we did not demonstrate the localization of TRPC3 in ECs. However, Senadheera et al (2012) [41] showed the loss of TRPC3 in ECs of TRPC3 KO mice generated by same protocol with the present study. They demonstrated the presence of TRPC3 in aortic endothelium and smooth muscle using TRPC3 antibody confocal immunohistochemistry. They also demonstrated TRPC3 was absent in both cell layers of vessels from TRPC3 KO mouse. Therefore, the inhibition of endothelium-dependent vasorelaxation in TRPC3 KO mice may be due to decrease in Ca^2+^ influx via TRPC3 channels of ECs. These results suggest that TRPC3 participates in endothelial cell Ca^2+^ influx and NO production.

Ca^2+^ influx through plasma membrane Ca^2+^-permeable channels plays an important role in endothelial cell physiology and changes in intracellular Ca^2+^ concentration associated to receptor-operated Ca^2+^ entry have a profound impact on diverse endothelial function [42]. TRPC channels are now recognized among the most important Ca^2+^-permeable channels in vascular endothelium and the endothelium TRPC3-mediated Ca^2+^ influx may contributes to release of NO [12]. However, TRPC3 is activated downstream phosphoinositide-specific phospholipase C and can mediate SOCE and non-SOCE (second messenger-operated Ca^2+^ channels and receptor-operated Ca^2+^ channels) under physiological conditions of receptor stimulation by acetylcholine [42]. In the present study, therefore, it is unclear which mechanism between SOCE and non-SOCE involve in NO production via endothelium TRPC3-mediated Ca^2+^ influx. Pyrazole-3 may can inhibits receptor-operated Ca^2+^ channels as well as SOCE channels [43]. To determine if the SOCE mechanism involved in TRPC3-mediated Ca^2+^ influx, in the present study, we tested the effect of pyrazole-3 on SOCE using SERCA inhibitor thapsigargin. Although the peak of SOCE was partially reduced, but not significantly, in the presence of pyrazole-3 and the slope of SOCE were similar in the absence and presence of pyrazole-3. Therefore, TRPC3 may plays role in NO-mediated vasorelaxation as a receptor mediated Ca^2+^ channels in endothelial cells. However, the function of TRPC3 as part of SOCE channels was not fully ruled out and remains to be elucidated.

In summary, the present study directly demonstrated that the TRPC3 channel functions in agonist-induced vasoconstriction and plays a major role in NO-mediated vasorelaxation of intact mesenteric arteries.
